# 2-(8-Bromo­imidazo[1,2-*a*]pyridin-2-yl)-*N*′-[(*E*)-4-diethyl­amino-2-hy­droxy­benzyl­idene]acetohydrazide dihydrate

**DOI:** 10.1107/S160053681200685X

**Published:** 2012-02-24

**Authors:** Hoong-Kun Fun, Wan-Sin Loh, Seema Shenvi, Arun M. Isloor, Gurumurthy Hegde

**Affiliations:** a X-ray Crystallography Unit, School of Physics, Universiti Sains Malaysia, 11800 USM, Penang, Malaysia; b Organic Electronics Division, Department of Chemistry, National Institute of Technology - Karnataka, Surathkal, Mangalore 575 025, India; c Faculty of Industrial Science and Technology, Universiti Malaysia Pahang, Lebuhraya Tun Razak, 26300 Gambang, Kuantan, Pahang Darul Makmur, Malaysia

## Abstract

In the title compound, C_20_H_22_BrN_5_O_2_·2H_2_O, the Schiff base mol­ecule exists in an *E* conformation with respect to the acyclic C=N bond. An *S*(6) ring motif is formed *via* an intra­molecular O—H⋯N hydrogen bond. The dihedral angle between the imidazo[1,2-*a*]pyridine system and the benzene ring is 84.62 (5)°. In the crystal, N—H⋯O, O—H⋯O, O—H⋯N, C—H⋯O and C—H⋯Br hydrogen bonds link the mol­ecules into a three-dimensional network. The crystal packing is further stabilized by C—H⋯π and π–π inter­actions [centroid–centroid distance = 3.5365 (7) Å].

## Related literature

For background to and applications of hydrazones, see: Seleem *et al.* (2011 ▶); Rollas & Küçükgüzel (2007 ▶). For background to and applications of imidazopyridine, see: Ertepinarl *et al.* (1995 ▶); Liang *et al.* (2007 ▶); Hamdouchi *et al.* (1999 ▶); Gudmundsson & Johns (2007 ▶); Biftu *et al.* (2006 ▶); Fisher & Lusi (1972 ▶); Bochis *et al.* (1981 ▶). For hydrogen-bond motifs, see: Bernstein *et al.* (1995 ▶). For the stability of the temperature controller used for the data collection, see: Cosier & Glazer (1986 ▶).
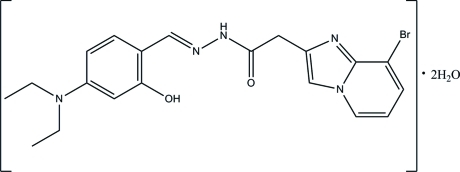


## Experimental

### 

#### Crystal data

C_20_H_22_BrN_5_O_2_·2H_2_O
*M* *_r_* = 480.37
Triclinic, 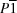

*a* = 8.4370 (4) Å
*b* = 10.6711 (5) Å
*c* = 11.7559 (5) Å
α = 92.914 (1)°
β = 96.949 (1)°
γ = 93.978 (1)°
*V* = 1046.23 (8) Å^3^
*Z* = 2
Mo *K*α radiation
μ = 2.00 mm^−1^
*T* = 100 K
0.37 × 0.20 × 0.07 mm

#### Data collection

Bruker SMART APEXII CCD diffractometer
Absorption correction: multi-scan (*SADABS*; Bruker, 2009 ▶) *T*_min_ = 0.529, *T*_max_ = 0.869
22937 measured reflections
6491 independent reflections
5841 reflections with *I* > 2σ(*I*)
*R*_int_ = 0.023

#### Refinement

*R*[*F* ^2^ > 2σ(*F* ^2^)] = 0.027
*wR*(*F* ^2^) = 0.077
*S* = 1.05
6491 reflections
297 parameters
H atoms treated by a mixture of independent and constrained refinement
Δρ_max_ = 0.63 e Å^−3^
Δρ_min_ = −0.24 e Å^−3^

### 

Data collection: *APEX2* (Bruker, 2009 ▶); cell refinement: *SAINT* (Bruker, 2009 ▶); data reduction: *SAINT*; program(s) used to solve structure: *SHELXTL* (Sheldrick, 2008 ▶); program(s) used to refine structure: *SHELXTL*; molecular graphics: *SHELXTL*; software used to prepare material for publication: *SHELXTL* and *PLATON* (Spek, 2009 ▶).

## Supplementary Material

Crystal structure: contains datablock(s) global, I. DOI: 10.1107/S160053681200685X/bq2340sup1.cif

Structure factors: contains datablock(s) I. DOI: 10.1107/S160053681200685X/bq2340Isup2.hkl

Supplementary material file. DOI: 10.1107/S160053681200685X/bq2340Isup3.cml

Additional supplementary materials:  crystallographic information; 3D view; checkCIF report

## Figures and Tables

**Table 1:** Hydrogen-bond geometry (Å, °) *Cg*1 is the centroid of the N1/C1/N2/C6/C7 ring and *Cg*3 is the centroid of the C11–C16 ring.

*D*—H⋯*A*	*D*—H	H⋯*A*	*D*⋯*A*	*D*—H⋯*A*
N3—H1N3⋯O2*W*^i^	0.894 (17)	1.908 (17)	2.7956 (15)	171.7 (17)
O2—H1O2⋯O1*W*^ii^	0.87 (3)	2.42 (3)	2.9423 (15)	119 (2)
O2—H1O2⋯N4	0.87 (3)	1.99 (3)	2.7181 (16)	142 (2)
O1*W*—H1*W*1⋯N1	0.86 (2)	1.98 (2)	2.8315 (14)	176 (2)
O1*W*—H2*W*1⋯O1^iii^	0.85 (3)	1.92 (2)	2.7361 (14)	162 (2)
O2*W*—H1*W*2⋯O1*W*^i^	0.80 (2)	2.08 (2)	2.8311 (15)	157 (2)
O2*W*—H2*W*2⋯O1*W*	0.86 (2)	1.87 (2)	2.7245 (15)	172.9 (17)
C5—H5*A*⋯O1^iv^	0.93	2.50	3.3121 (17)	146
C10—H10*A*⋯O2*W*^i^	0.93	2.54	3.3256 (17)	142
C17—H17*B*⋯Br1^v^	0.97	2.85	3.6569 (15)	142
C3—H3*A*⋯*Cg*3^vi^	0.93	2.61	3.4734 (15)	154
C17—H17*A*⋯*Cg*1^vii^	0.97	2.70	3.5863 (15)	152

Symmetry codes: (i) 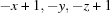; (ii) 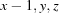; (iii) 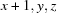; (iv) 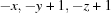; (v) 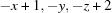; (vi) 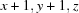; (vii) 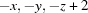.

**Fig. 1.**
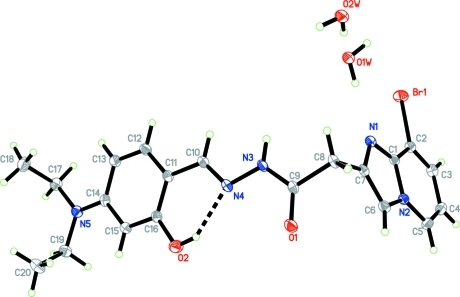
The molecular structure of the title compound, showing 50% probability displacement ellipsoids. Dashed line indicates the intramolecular hydrogen bond.

**Fig. 2.**
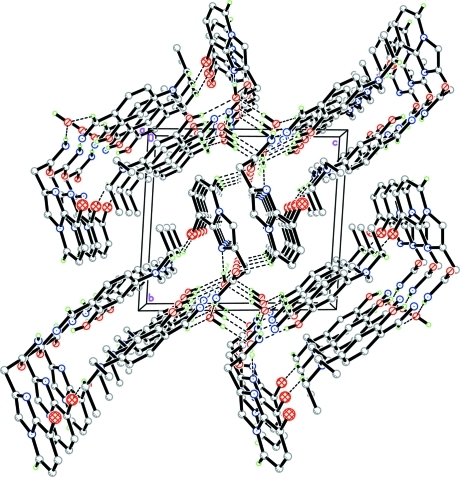
The crystal packing of the title compound, viewed along the a axis, showing the three-dimensional network. H atoms not involved in the intermolecular interactions (dashed lines) have been omitted for clarity.

## supplementary crystallographic information

### Comment

Hydrazones constitute an important class of biologically active drug molecules (Seleem *et al.*, 2011) which has attracted attention of medicinal chemists due to their wide range of pharmacological properties. These compounds are being synthesized as drugs by many researchers in order to combat diseases with minimal toxicity and maximal effects. A number of hydrazone derivatives have been reported to exert notably antimicrobial, antihypertensive, anticonvulsant, analgesic, anti-inflammatory, antituberculosis, antitumoral, antiproliferative and antimalarial activities (Rollas & Küçükgüzel, 2007). Imidazopyridine is the fundamental heterocyclic component of principal anthelmintic drugs. In addition, the imidazopyridine ring possesses many anti-infective properties including antibacterial (Ertepinarl *et al.*, 1995; Liang *et al.*, 2007), antiviral (Hamdouchi *et al.*, 1999; Gudmundsson & Johns, 2007), antiprotozoal (Biftu *et al.*, 2006) and especially anthelmintic (Fisher & Lusi, 1972; Bochis *et al.*, 1981) activities. Therefore, the imidazopyridine ring could replace the benzimidazole ring in the design and the development of new anthelmintic agents. In view of its biological importance, we hereby report the crystal structure of (I).

The title compound (Fig. 1) consists of one 2-(8-bromoimidazo[1,2-*a*]pyridin-3-yl)-*N*'-{(*E*)- [4-(diethylamino)-2-hydroxyphenyl]methylidene}acetohydrazide molecule and two water molecules. The Schiff base molecule exists in an *E* configuration with respect to the acyclic C═N bond. An *S*(6) ring motif (Bernstein *et al.*, 1995) is formed *via* the intramolecular O2—H1O2···N4 hydrogen bond. The dihedral angle between the imidazo[1,2-*a*]pyridine (C1–C5/N2/C6/C7/N1) and the benzene (C11–C16) rings is 84.62 (5)°.

In the crystal packing (Fig. 2), intermolecular N3—H1N3···O2W, O2—H1O2···O1W, O2—H1O2···N4, O1W—H1W1···N1, O1W—H2W1···O1, O2W—H1W2···O1W, O2W—H2W2···O1W, C5—H5A···O1, C10—H10A···O2W and C17—H17B···Br1 hydrogen bonds link the molecules into a three-dimensional network. The crystal packing is further stabilized by C—H···π interactions, involving the 1*H*-imidazole (N1/C1/N2/C6/C7; *Cg*1; Table 1) and benzene (*Cg*3; Table 1) rings. Weak π–π interactions were observed involving 1*H*-imidazole, pyridine (N2/C1–C5; *Cg*2) and benzene rings. [*Cg*1···*Cg*1 = 3.5365 (7) Å; symmetry code: 1 - *x*, 1 - *y*, 1 - *z*; *Cg*1···*Cg*2 = 3.6210 (7) Å; symmetry code: 1 - *x*, 1 - *y*, 1 - *z*; *Cg*3···*Cg*3 = 3.6253 (8) Å; symmetry code: -*x*, -*y*, 2 - *z*].

### Experimental

The mixture of 2-(8-bromoimidazo[1,2-*a*]pyridine-3-yl)acetohydrazide (200 mg, 0.00074 mol), 4-(diethylamino)-2-hydroxy benzaldehyde (143.6 mg, 0.00074 mol) and a catalytic quantity of acetic acid (0.1 ml) and ethanol (10 ml) was stirred overnight at 90°C. On cooling, orange plate-shaped crystals of the product begins to separate. It was collected by filtration and recrystallized from ethanol. Yield: 307.7 mg, 93.2%. m.p. 401–402 K.

### Refinement

O- and N-bound H atoms were located from the difference Fourier map and were refined freely [O—H = 0.80 (3) to 0.87 (3) Å; N—H = 0.896 (19) Å]. The remaining H atoms were positioned geometrically and refined with a riding model with *U*_iso_(H) = 1.2 or 1.5*U*_eq_(C) [C—H = 0.93 to 0.97 Å]. A rotating group model was applied to the methyl groups. Three outliners were omitted in the final refinement, 230, 541 and 365.

### Crystal data

**Table:**

C_20_H_22_BrN_5_O_2_·2H_2_O	*Z* = 2
*M**_r_* = 480.37	*F*(000) = 496
Triclinic, *P*1	*D*_x_ = 1.525 Mg m^−^^3^
Hall symbol: -P 1	Mo *K*α radiation, λ = 0.71073 Å
*a* = 8.4370 (4) Å	Cell parameters from 9983 reflections
*b* = 10.6711 (5) Å	θ = 2.4–30.9°
*c* = 11.7559 (5) Å	µ = 2.00 mm^−^^1^
α = 92.914 (1)°	*T* = 100 K
β = 96.949 (1)°	Plate, orange
γ = 93.978 (1)°	0.37 × 0.20 × 0.07 mm
*V* = 1046.23 (8) Å^3^	

### Data collection

**Table:**

Bruker SMART APEXII CCD diffractometer	6491 independent reflections
Radiation source: fine-focus sealed tube	5841 reflections with *I* > 2σ(*I*)
Graphite monochromator	*R*_int_ = 0.023
φ and ω scans	θ_max_ = 30.9°, θ_min_ = 1.8°
Absorption correction: multi-scan (*SADABS*; Bruker, 2009)	*h* = −12→12
*T*_min_ = 0.529, *T*_max_ = 0.869	*k* = −15→15
22937 measured reflections	*l* = −16→16

### Refinement

**Table:**

Refinement on *F*^2^	Primary atom site location: structure-invariant direct methods
Least-squares matrix: full	Secondary atom site location: difference Fourier map
*R*[*F*^2^ > 2σ(*F*^2^)] = 0.027	Hydrogen site location: inferred from neighbouring sites
*wR*(*F*^2^) = 0.077	H atoms treated by a mixture of independent and constrained refinement
*S* = 1.05	*w* = 1/[σ^2^(*F*_o_^2^) + (0.0455*P*)^2^ + 0.2952*P*] where *P* = (*F*_o_^2^ + 2*F*_c_^2^)/3
6491 reflections	(Δ/σ)_max_ = 0.002
297 parameters	Δρ_max_ = 0.63 e Å^−^^3^
0 restraints	Δρ_min_ = −0.24 e Å^−^^3^

### Special details

**Table:**

Experimental. The crystal was placed in the cold stream of an Oxford Cryosystems Cobra open-flow nitrogen cryostat (Cosier & Glazer, 1986) operating at 100.0 (1) K.
Geometry. All e.s.d.'s (except the e.s.d. in the dihedral angle between two l.s. planes) are estimated using the full covariance matrix. The cell e.s.d.'s are taken into account individually in the estimation of e.s.d.'s in distances, angles and torsion angles; correlations between e.s.d.'s in cell parameters are only used when they are defined by crystal symmetry. An approximate (isotropic) treatment of cell e.s.d.'s is used for estimating e.s.d.'s involving l.s. planes.
Refinement. Refinement of *F*^2^ against ALL reflections. The weighted *R*-factor *wR* and goodness of fit *S* are based on *F*^2^, conventional *R*-factors *R* are based on *F*, with *F* set to zero for negative *F*^2^. The threshold expression of *F*^2^ > σ(*F*^2^) is used only for calculating *R*-factors(gt) *etc*. and is not relevant to the choice of reflections for refinement. *R*-factors based on *F*^2^ are statistically about twice as large as those based on *F*, and *R*-factors based on ALL data will be even larger.

### Fractional atomic coordinates and isotropic or equivalent isotropic displacement parameters (Å^2^)

**Table:**

	*x*	*y*	*z*	*U*_iso_*/*U*_eq_	
Br1	0.789019 (15)	0.424051 (13)	0.748329 (12)	0.02141 (5)	
O1	−0.05933 (11)	0.23815 (10)	0.52050 (9)	0.02047 (19)	
O2	−0.26058 (13)	0.04472 (10)	0.79255 (9)	0.0227 (2)	
N1	0.44340 (12)	0.35221 (10)	0.59413 (9)	0.01348 (19)	
N2	0.35279 (12)	0.54369 (10)	0.61740 (9)	0.01344 (19)	
N3	0.10753 (13)	0.09994 (10)	0.60467 (9)	0.01444 (19)	
N4	−0.00110 (13)	0.05569 (10)	0.67606 (9)	0.0150 (2)	
N5	−0.25929 (13)	−0.22618 (11)	1.10154 (9)	0.0164 (2)	
C1	0.47756 (14)	0.46769 (11)	0.64404 (10)	0.0127 (2)	
C2	0.61422 (15)	0.52232 (12)	0.71616 (11)	0.0147 (2)	
C3	0.62011 (16)	0.64472 (13)	0.75761 (11)	0.0174 (2)	
H3A	0.7093	0.6799	0.8059	0.021*	
C4	0.48898 (17)	0.71799 (12)	0.72639 (11)	0.0182 (2)	
H4A	0.4935	0.8015	0.7542	0.022*	
C5	0.35760 (16)	0.66812 (12)	0.65681 (11)	0.0175 (2)	
H5A	0.2725	0.7167	0.6361	0.021*	
C6	0.23518 (14)	0.47094 (12)	0.54616 (11)	0.0153 (2)	
H6A	0.1371	0.4957	0.5135	0.018*	
C7	0.29303 (14)	0.35486 (12)	0.53359 (10)	0.0134 (2)	
C8	0.20850 (15)	0.23836 (12)	0.46996 (11)	0.0155 (2)	
H8A	0.2831	0.1737	0.4649	0.019*	
H8B	0.1674	0.2574	0.3926	0.019*	
C9	0.07088 (14)	0.19108 (12)	0.53330 (11)	0.0143 (2)	
C10	0.05212 (15)	−0.02976 (12)	0.74143 (11)	0.0158 (2)	
H10A	0.1519	−0.0582	0.7326	0.019*	
C11	−0.03602 (15)	−0.08324 (12)	0.82756 (11)	0.0146 (2)	
C12	0.03051 (15)	−0.17581 (13)	0.89465 (12)	0.0175 (2)	
H12A	0.1274	−0.2048	0.8788	0.021*	
C13	−0.04169 (15)	−0.22574 (12)	0.98328 (11)	0.0173 (2)	
H13A	0.0055	−0.2883	1.0247	0.021*	
C14	−0.18834 (15)	−0.18158 (12)	1.01136 (10)	0.0144 (2)	
C15	−0.25764 (15)	−0.08989 (12)	0.94303 (11)	0.0156 (2)	
H15A	−0.3549	−0.0610	0.9582	0.019*	
C16	−0.18382 (15)	−0.04205 (12)	0.85370 (11)	0.0147 (2)	
C17	−0.19118 (17)	−0.32564 (13)	1.16815 (12)	0.0196 (2)	
H17A	−0.2326	−0.3243	1.2416	0.024*	
H17B	−0.0760	−0.3076	1.1831	0.024*	
C18	−0.2262 (2)	−0.45760 (14)	1.11027 (14)	0.0265 (3)	
H18A	−0.1691	−0.5164	1.1555	0.040*	
H18B	−0.1926	−0.4586	1.0351	0.040*	
H18C	−0.3391	−0.4808	1.1038	0.040*	
C19	−0.41481 (16)	−0.18615 (13)	1.12605 (11)	0.0186 (2)	
H19A	−0.4149	−0.0958	1.1202	0.022*	
H19B	−0.4286	−0.2039	1.2045	0.022*	
C20	−0.55683 (17)	−0.25000 (16)	1.04598 (13)	0.0252 (3)	
H20A	−0.6542	−0.2176	1.0652	0.038*	
H20B	−0.5615	−0.3391	1.0544	0.038*	
H20C	−0.5440	−0.2334	0.9680	0.038*	
O1W	0.64543 (12)	0.15365 (9)	0.57170 (9)	0.01893 (19)	
O2W	0.60246 (12)	0.01353 (10)	0.36855 (10)	0.01989 (19)	
H1N3	0.205 (2)	0.0710 (17)	0.6120 (15)	0.018 (4)*	
H1O2	−0.209 (3)	0.069 (3)	0.737 (2)	0.058 (8)*	
H1W1	0.587 (3)	0.215 (2)	0.582 (2)	0.041 (6)*	
H2W1	0.742 (3)	0.181 (2)	0.5720 (18)	0.031 (5)*	
H1W2	0.518 (3)	−0.026 (2)	0.369 (2)	0.041 (6)*	
H2W2	0.608 (2)	0.060 (2)	0.4315 (18)	0.026 (5)*	

### Atomic displacement parameters (Å^2^)

**Table:**

	*U*^11^	*U*^22^	*U*^33^	*U*^12^	*U*^13^	*U*^23^
Br1	0.01455 (7)	0.02522 (8)	0.02311 (8)	0.00475 (5)	−0.00432 (5)	−0.00016 (5)
O1	0.0123 (4)	0.0243 (5)	0.0255 (5)	0.0016 (3)	0.0027 (4)	0.0083 (4)
O2	0.0220 (5)	0.0254 (5)	0.0236 (5)	0.0075 (4)	0.0069 (4)	0.0106 (4)
N1	0.0117 (4)	0.0156 (5)	0.0132 (5)	0.0004 (4)	0.0019 (4)	0.0016 (4)
N2	0.0121 (4)	0.0152 (5)	0.0133 (5)	0.0009 (3)	0.0018 (4)	0.0027 (4)
N3	0.0107 (4)	0.0166 (5)	0.0165 (5)	−0.0009 (4)	0.0041 (4)	0.0021 (4)
N4	0.0126 (4)	0.0178 (5)	0.0147 (5)	−0.0017 (4)	0.0037 (4)	0.0009 (4)
N5	0.0165 (5)	0.0194 (5)	0.0136 (5)	0.0012 (4)	0.0030 (4)	0.0031 (4)
C1	0.0115 (5)	0.0154 (5)	0.0115 (5)	0.0013 (4)	0.0022 (4)	0.0031 (4)
C2	0.0130 (5)	0.0177 (6)	0.0131 (5)	0.0007 (4)	0.0007 (4)	0.0020 (4)
C3	0.0179 (6)	0.0194 (6)	0.0138 (5)	−0.0021 (4)	0.0005 (4)	0.0001 (4)
C4	0.0229 (6)	0.0151 (5)	0.0167 (6)	0.0017 (4)	0.0036 (5)	0.0013 (4)
C5	0.0199 (6)	0.0156 (6)	0.0178 (6)	0.0040 (4)	0.0036 (5)	0.0036 (4)
C6	0.0109 (5)	0.0197 (6)	0.0148 (5)	0.0000 (4)	−0.0001 (4)	0.0026 (4)
C7	0.0118 (5)	0.0171 (5)	0.0115 (5)	−0.0009 (4)	0.0030 (4)	0.0024 (4)
C8	0.0136 (5)	0.0188 (6)	0.0140 (5)	−0.0030 (4)	0.0039 (4)	0.0004 (4)
C9	0.0122 (5)	0.0160 (5)	0.0141 (5)	−0.0030 (4)	0.0017 (4)	0.0001 (4)
C10	0.0129 (5)	0.0173 (6)	0.0169 (6)	−0.0007 (4)	0.0024 (4)	0.0003 (4)
C11	0.0131 (5)	0.0164 (5)	0.0139 (5)	−0.0013 (4)	0.0010 (4)	0.0009 (4)
C12	0.0132 (5)	0.0200 (6)	0.0193 (6)	0.0014 (4)	0.0017 (4)	0.0029 (5)
C13	0.0154 (5)	0.0187 (6)	0.0176 (6)	0.0012 (4)	0.0004 (4)	0.0041 (4)
C14	0.0155 (5)	0.0152 (5)	0.0113 (5)	−0.0022 (4)	−0.0003 (4)	−0.0002 (4)
C15	0.0145 (5)	0.0173 (6)	0.0152 (6)	0.0010 (4)	0.0023 (4)	0.0007 (4)
C16	0.0144 (5)	0.0150 (5)	0.0144 (5)	−0.0004 (4)	0.0008 (4)	0.0007 (4)
C17	0.0227 (6)	0.0207 (6)	0.0158 (6)	0.0017 (5)	0.0024 (5)	0.0040 (5)
C18	0.0312 (8)	0.0201 (7)	0.0288 (8)	0.0007 (5)	0.0058 (6)	0.0029 (5)
C19	0.0193 (6)	0.0230 (6)	0.0145 (6)	0.0023 (5)	0.0055 (5)	0.0016 (5)
C20	0.0177 (6)	0.0342 (8)	0.0235 (7)	−0.0006 (5)	0.0040 (5)	−0.0005 (6)
O1W	0.0137 (4)	0.0173 (4)	0.0263 (5)	0.0007 (3)	0.0043 (4)	0.0026 (4)
O2W	0.0150 (4)	0.0182 (4)	0.0270 (5)	0.0005 (3)	0.0057 (4)	0.0000 (4)

### Geometric parameters (Å, º)

**Table:**

Br1—C2	1.8809 (13)	C8—H8B	0.9700
O1—C9	1.2354 (16)	C10—C11	1.4444 (17)
O2—C16	1.3534 (16)	C10—H10A	0.9300
O2—H1O2	0.87 (3)	C11—C12	1.4007 (18)
N1—C1	1.3350 (16)	C11—C16	1.4134 (17)
N1—C7	1.3804 (16)	C12—C13	1.3804 (18)
N2—C5	1.3802 (16)	C12—H12A	0.9300
N2—C6	1.3837 (16)	C13—C14	1.4232 (18)
N2—C1	1.3887 (15)	C13—H13A	0.9300
N3—C9	1.3443 (17)	C14—C15	1.4108 (17)
N3—N4	1.3906 (14)	C15—C16	1.3856 (18)
N3—H1N3	0.896 (19)	C15—H15A	0.9300
N4—C10	1.2915 (17)	C17—C18	1.526 (2)
N5—C14	1.3690 (16)	C17—H17A	0.9700
N5—C17	1.4594 (17)	C17—H17B	0.9700
N5—C19	1.4642 (17)	C18—H18A	0.9600
C1—C2	1.4170 (17)	C18—H18B	0.9600
C2—C3	1.3662 (18)	C18—H18C	0.9600
C3—C4	1.4216 (19)	C19—C20	1.528 (2)
C3—H3A	0.9300	C19—H19A	0.9700
C4—C5	1.3548 (19)	C19—H19B	0.9700
C4—H4A	0.9300	C20—H20A	0.9600
C5—H5A	0.9300	C20—H20B	0.9600
C6—C7	1.3701 (17)	C20—H20C	0.9600
C6—H6A	0.9300	O1W—H1W1	0.86 (3)
C7—C8	1.5057 (17)	O1W—H2W1	0.84 (2)
C8—C9	1.5242 (17)	O2W—H1W2	0.80 (3)
C8—H8A	0.9700	O2W—H2W2	0.86 (2)
			
C16—O2—H1O2	111.7 (18)	C12—C11—C16	116.99 (11)
C1—N1—C7	105.16 (10)	C12—C11—C10	119.37 (11)
C5—N2—C6	130.13 (11)	C16—C11—C10	123.53 (12)
C5—N2—C1	123.09 (11)	C13—C12—C11	122.76 (12)
C6—N2—C1	106.77 (10)	C13—C12—H12A	118.6
C9—N3—N4	120.60 (10)	C11—C12—H12A	118.6
C9—N3—H1N3	121.0 (12)	C12—C13—C14	120.09 (12)
N4—N3—H1N3	118.2 (12)	C12—C13—H13A	120.0
C10—N4—N3	113.55 (10)	C14—C13—H13A	120.0
C14—N5—C17	120.87 (11)	N5—C14—C15	121.23 (11)
C14—N5—C19	121.09 (11)	N5—C14—C13	121.24 (12)
C17—N5—C19	117.68 (11)	C15—C14—C13	117.54 (11)
N1—C1—N2	111.09 (10)	C16—C15—C14	121.34 (12)
N1—C1—C2	131.60 (11)	C16—C15—H15A	119.3
N2—C1—C2	117.31 (11)	C14—C15—H15A	119.3
C3—C2—C1	120.47 (12)	O2—C16—C15	116.85 (11)
C3—C2—Br1	121.18 (10)	O2—C16—C11	121.89 (12)
C1—C2—Br1	118.31 (9)	C15—C16—C11	121.25 (12)
C2—C3—C4	119.53 (12)	N5—C17—C18	114.42 (12)
C2—C3—H3A	120.2	N5—C17—H17A	108.7
C4—C3—H3A	120.2	C18—C17—H17A	108.7
C5—C4—C3	121.06 (12)	N5—C17—H17B	108.7
C5—C4—H4A	119.5	C18—C17—H17B	108.7
C3—C4—H4A	119.5	H17A—C17—H17B	107.6
C4—C5—N2	118.52 (12)	C17—C18—H18A	109.5
C4—C5—H5A	120.7	C17—C18—H18B	109.5
N2—C5—H5A	120.7	H18A—C18—H18B	109.5
C7—C6—N2	105.77 (11)	C17—C18—H18C	109.5
C7—C6—H6A	127.1	H18A—C18—H18C	109.5
N2—C6—H6A	127.1	H18B—C18—H18C	109.5
C6—C7—N1	111.22 (11)	N5—C19—C20	113.89 (11)
C6—C7—C8	127.58 (11)	N5—C19—H19A	108.8
N1—C7—C8	121.11 (11)	C20—C19—H19A	108.8
C7—C8—C9	109.35 (10)	N5—C19—H19B	108.8
C7—C8—H8A	109.8	C20—C19—H19B	108.8
C9—C8—H8A	109.8	H19A—C19—H19B	107.7
C7—C8—H8B	109.8	C19—C20—H20A	109.5
C9—C8—H8B	109.8	C19—C20—H20B	109.5
H8A—C8—H8B	108.3	H20A—C20—H20B	109.5
O1—C9—N3	124.94 (11)	C19—C20—H20C	109.5
O1—C9—C8	120.90 (12)	H20A—C20—H20C	109.5
N3—C9—C8	114.12 (11)	H20B—C20—H20C	109.5
N4—C10—C11	123.10 (12)	H1W1—O1W—H2W1	110 (2)
N4—C10—H10A	118.5	H1W2—O2W—H2W2	101 (2)
C11—C10—H10A	118.5		
			
C9—N3—N4—C10	−178.57 (11)	C7—C8—C9—O1	81.70 (15)
C7—N1—C1—N2	0.28 (13)	C7—C8—C9—N3	−95.99 (13)
C7—N1—C1—C2	−179.63 (13)	N3—N4—C10—C11	175.89 (11)
C5—N2—C1—N1	−179.26 (11)	N4—C10—C11—C12	179.87 (12)
C6—N2—C1—N1	−0.44 (13)	N4—C10—C11—C16	−4.1 (2)
C5—N2—C1—C2	0.66 (17)	C16—C11—C12—C13	−0.39 (19)
C6—N2—C1—C2	179.48 (11)	C10—C11—C12—C13	175.88 (12)
N1—C1—C2—C3	−179.62 (13)	C11—C12—C13—C14	−1.3 (2)
N2—C1—C2—C3	0.47 (18)	C17—N5—C14—C15	176.84 (12)
N1—C1—C2—Br1	2.65 (19)	C19—N5—C14—C15	3.84 (18)
N2—C1—C2—Br1	−177.26 (8)	C17—N5—C14—C13	−3.34 (18)
C1—C2—C3—C4	−1.02 (19)	C19—N5—C14—C13	−176.34 (12)
Br1—C2—C3—C4	176.64 (10)	C12—C13—C14—N5	−177.55 (12)
C2—C3—C4—C5	0.5 (2)	C12—C13—C14—C15	2.28 (19)
C3—C4—C5—N2	0.6 (2)	N5—C14—C15—C16	178.18 (12)
C6—N2—C5—C4	−179.72 (12)	C13—C14—C15—C16	−1.64 (18)
C1—N2—C5—C4	−1.20 (19)	C14—C15—C16—O2	179.42 (11)
C5—N2—C6—C7	179.12 (12)	C14—C15—C16—C11	−0.03 (19)
C1—N2—C6—C7	0.41 (13)	C12—C11—C16—O2	−178.35 (12)
N2—C6—C7—N1	−0.25 (14)	C10—C11—C16—O2	5.54 (19)
N2—C6—C7—C8	176.21 (11)	C12—C11—C16—C15	1.06 (18)
C1—N1—C7—C6	−0.01 (14)	C10—C11—C16—C15	−175.04 (12)
C1—N1—C7—C8	−176.73 (11)	C14—N5—C17—C18	−78.07 (16)
C6—C7—C8—C9	−68.28 (16)	C19—N5—C17—C18	95.16 (14)
N1—C7—C8—C9	107.87 (13)	C14—N5—C19—C20	77.22 (16)
N4—N3—C9—O1	−3.51 (19)	C17—N5—C19—C20	−96.00 (14)
N4—N3—C9—C8	174.06 (10)		

### Hydrogen-bond geometry (Å, º)

Cg1 is the centroid of the N1/C1/N2/C6/C7 ring and Cg3 is the centroid of the C11–C16 ring.

**Table:**

*D*—H···*A*	*D*—H	H···*A*	*D*···*A*	*D*—H···*A*
N3—H1*N*3···O2*W*^i^	0.894 (17)	1.908 (17)	2.7956 (15)	171.7 (17)
O2—H1*O*2···O1*W*^ii^	0.87 (3)	2.42 (3)	2.9423 (15)	119 (2)
O2—H1*O*2···N4	0.87 (3)	1.99 (3)	2.7181 (16)	142 (2)
O1*W*—H1*W*1···N1	0.86 (2)	1.98 (2)	2.8315 (14)	176 (2)
O1*W*—H2*W*1···O1^iii^	0.85 (3)	1.92 (2)	2.7361 (14)	162 (2)
O2*W*—H1*W*2···O1*W*^i^	0.80 (2)	2.08 (2)	2.8311 (15)	157 (2)
O2*W*—H2*W*2···O1*W*	0.86 (2)	1.87 (2)	2.7245 (15)	172.9 (17)
C5—H5*A*···O1^iv^	0.93	2.50	3.3121 (17)	146
C10—H10*A*···O2*W*^i^	0.93	2.54	3.3256 (17)	142
C17—H17*B*···Br1^v^	0.97	2.85	3.6569 (15)	142
C3—H3*A*···*Cg*3^vi^	0.93	2.61	3.4734 (15)	154
C17—H17*A*···*Cg*1^vii^	0.97	2.70	3.5863 (15)	152

Symmetry codes: (i) −*x*+1, −*y*, −*z*+1; (ii) *x*−1, *y*, *z*; (iii) *x*+1, *y*, *z*; (iv) −*x*, −*y*+1, −*z*+1; (v) −*x*+1, −*y*, −*z*+2; (vi) *x*+1, *y*+1, *z*; (vii) −*x*, −*y*, −*z*+2.
